# The Role of the General Practitioner

**Published:** 1913-06-14

**Authors:** H. Hyslop Thomson

**Affiliations:** Tuberculosis Officer for the County of Herts; late Medical Superintendent of the Liverpool Sanatorium.


### MODERN CAMPAIGN AGAINST TUBERCULOSIS.

### II.—The Role of the General Practitioner.

By H. HYSLOP THOMSON, M.D., D.P.H., Tuberculosis Officer for the County of Herts; late Medical Superintendent of the Liverpool Sanatorium.

It has frequently been suggested that the institution of the new service of tuberculosis officer will narrow down the sphere of work and diminish the responsibility of the general practitioner in relation to tuberculosis. Although this may be so to some extent, it is in many respects more apparent than real, and, indeed, in some directions it will be found that, as the schemes for dealing with tuberculosis throughout the country become organised, the duties and responsibilities of the general practitioner in relation thereto will become increased. Previous to the passing of the National Insurance Act the treatment of tuberculosis was carried on throughout the country in a haphazard, disjointed fashion, without any uniform method of procedure. The general practitioner had to carry on an unequal fight against the disease, in that he was compelled to treat many of his patients in their homes under most unsuitable conditions, and only in a small percentage of cases was he able to secure suitable sanatorium or dispensary treatment. Now, however, that local authorities are organising schemes for the treatment of tuberculosis the difficulties with which the general practitioner had formerly to contend will gradually be removed, and the important position he occupies in the modern campaign against the disease will become more clearly defined.

### The General Practitioner and Diagnosis.

Success in the treatment of tuberculosis rests on early diagnosis, and it is to the general practitioner we must look for early recognition of the disease. As a result of National Insurance the insured will more readily seek medical advice when they become conscious of any slight departure from their normal state of health. In consequence those, in whom the tubercle bacilli have secured a foothold, will seek medical advice and treatment in the early stages of infection. Cases of early pulmonary tuberculosis will therefore more frequently come under the observation of the general practitioner, so that his responsibilities with regard to early diagnosis and favourable prognosis will be increased. Moreover, in the future the general practitioner will have a greater incentive to detect cases in the early stages of infection, for he will no longer be faced with the impossibility of securing suitable treatment for his patient, but will be able to obtain the method of treatment which will best suit individual requirements.

In general practice the diagnosis of early pulmonary tuberculosis must still depend in large measure on the personal history of the case and on stethoscopic examination. In many cases, although the disease is strongly suspected, the diagnosis will be difficult owing to the indefinite nature of the physical signs, but here the patient can be referred to the dispensary for some confirmatory test. The position which the general practitioner occupies in the modern campaign against tuberculosis is therefore an important one, as it is to him that we must chiefly look for the supply of suitable clinical material and for a gradual increase in the ratio of early to advanced cases.

As a result of the new *régime* the position of the general practitioner in relation to treatment will be altered to some extent. In those districts in which it is proposed to provide adequate accommodation for the segregation and treatment of advanced and acute cases the general practitioner will be relieved of no little anxiety and responsibility with regard to the domiciliary treatment of such cases under unfavourable conditions. Certain insured patients will necessarily be recommended for domiciliary treatment either from the commencement or after a course of sanatorium treatment, and such cases will be under the supervision and treatment of the general practitioner. In the case of private patients whose home conditions are suitable a short period of sanatorium treatment, chiefly for educational purposes, followed by treatment at home in a shelter, has certain advantages, especially in districts where sanatorium accommodation is limited. In the interim report of the Departmental Committee on Tuberculosis it is pointed out that home treatment may be in all essentials sanatorium treatment, the details of which will be carried out under the supervision of the general practitioner. Domiciliary treatment, including if necessary medicinal treatment, will also be required for those attending the dispensary. To divorce the one from the other would militate against the best results being obtained, as home supervision with regard to hygienic and dietetic treatment is absolutely essential during a course of tuberculin treatment.

### Dispensary and After-Care.

It is not possible to define accurately the position of the general practitioner in relation to the dispensary as this will vary in different districts. While the tuberculosis officer will be responsible for the work at the dispensary he will require, especially in certain rural districts, the assistance and co-operation of the general practitioner. Such assistance may be given voluntarily, or it may be placed on an official basis so that the general practitioners in rotation act as the deputy of the tuberculosis officer. Such an arrangement, if efficiently and harmoniously carried out, has much to commend it. It will make for co-ordination of effort, unification of the methods of treatment, and smooth working of the scheme generally. Moreover, the question of after-care demands close co-operation between the general practitioner and tuberculosis officer, and it is almost unnecessary to add that much of the success of the modern campaign against tuberculosis will depend upon the efficiency with which after-care is carried out. While the dispensary will be the centre for organising the scheme of after-care, it is to the general practitioner to whom we must look for that supervision and controlling influence in the patient's home which is essential to any effort at successful after-care. Indeed, the after-results of the modern methods of treating tuberculosis depend in no small degree on the extent to which the general practitioner co-operates with the tuberculosis officer and actively participates in the scheme. The very greatest importance attaches to this co-operation, and results of considerable value are to be anticipated.

The general practitioner has good cause for complaint with regard to the question of tuberculin treatment in general practice. He looks to the experts for advice as to the form of tuberculin to be used, the method of administration, and the cases most suitable for treatment, but he finds no agreement as to procedure or uniformity of practice. At the present time there are so many divergent views as to the value and methods of tuberculin treatment that a state of confusion must exist in the minds of many general practitioners. It is to be hoped that this will soon be remedied by the dispensary system, and that each dispensary will become a centre from which will emanate a uniform method of treatment with tuberculin. This can only be secured, however, by active co-operation between the general practitioner and tuberculosis officer and a frank exchange of views regarding the procedure of treatment. Personally the writer has found such co-operation and exchange of views of the very greatest value in carrying out his duties as tuberculosis officer. The suggestion has been made that the general practitioner should obtain from the dispensary the necessary dilutions of tuberculin for carrying out treatment in the case of patients who do not attend the dispensary, and while this would entail considerable additional work on the part of the tuberculosis officer it has much to recommend it. It would secure active co-operation and would, above all else, make for a uniform method of treatment in the district, and this from a statistical point of view alone is earnestly to be desired. The *rôle* of the general practitioner in the present-day campaign against tuberculosis is an important one, and the assistance which he is able to offer in relation to diagnosis and treatment is of the very greatest value, and every effort therefore should be made to co-ordinate such effective help with the work of the dispensary and the sanatorium.

